# Mini-hemagglutinin vaccination induces cross-reactive antibodies in pre-exposed NHP that protect mice against lethal influenza challenge

**DOI:** 10.1038/s41541-018-0063-7

**Published:** 2018-07-03

**Authors:** Joan E. M. van der Lubbe, Jeroen Huizingh, Johan W. A. Verspuij, Lisanne Tettero, Sonja P. R. Schmit-Tillemans, Petra Mooij, Daniella Mortier, Gerrit Koopman, Willy M. J. M. Bogers, Liesbeth Dekking, Wim Meijberg, Ted Kwaks, Boerries Brandenburg, Jeroen T. B. M. Tolboom, Hanneke Schuitemaker, Ramon Roozendaal, Harmjan Kuipers, Roland C. Zahn

**Affiliations:** 1 Janssen Vaccines and Prevention, Pharmaceutical Companies of Johnson and Johnson, Archimedesweg 4-6, 2333 CN Leiden, The Netherlands; 2 Department of Virology, Biomedical Primate Research Centre, P.O. Box 3306, 2280 GH Rijswijk, The Netherlands; 3 Present Address: Innovation Exchange Amsterdam, IXA Academic Medical Center, Meibergdreef 9, 1105 AZ Amsterdam, The Netherlands

## Abstract

Seasonal vaccines are currently the most effective countermeasure against influenza. However, seasonal vaccines are only effective against strains closely related to the influenza strains contained in the vaccine. Recently a new hemagglutinin (HA) stem-based antigen, the so-called “mini-HA”, has been shown to induce a cross-protective immune response in influenza-naive mice and non-human primates (NHP). However, prior exposure to influenza can have a profound effect on the immune response to subsequent influenza infection and the protective efficacy of vaccination. Here we show that mini-HA, compared to a trivalent influenza vaccine (TIV), elicits a broadened influenza-specific humoral immune response in NHP previously exposed to influenza. Serum transfer experiments showed that antibodies induced by both mini-HA and seasonal vaccine protected mice against lethal challenge with a H1N1 influenza strain heterologous to the H1 HA included in the TIV. However, antibodies elicited by mini-HA showed an additional benefit of protecting mice against lethal heterosubtypic H5N1 influenza challenge, associated with H5 HA-specific functional antibodies.

## Influenza: a novel vaccine offers broad protection

A vaccine candidate developed from a novel flu-based protein induces antibodies able to protect against multiple influenza strains. Alongside a team of Dutch researchers, Janssen’s Joan van der Lubbe created a vaccine, dubbed “mini-HA”, based on a recently discovered influenza protein that induced neutralizing antibodies to many related influenza virus strains in non-human primates. Mini-HA vaccination targeted a surface protein region that is highly conserved between influenza strains and offered protection regardless of previous exposure to the disease, which is known to greatly affect vaccine efficacy. The immune response elicited by the novel vaccine surpassed that of a standard seasonal influenza vaccine. Future research is now warranted to verify whether mini-HA can act as a universal seasonal flu vaccine, potentially even effective against emergent virus strains.

## Introduction

Currently the most effective countermeasure available against influenza is vaccination. Due to rapid accumulation of point mutations and reassortment the influenza virus evades the protective immune response elicited by prior infections or vaccination. As a consequence the strains included in the vaccine need yearly review. Influenza strains to be included in the vaccine are selected based on continuous surveillance of circulating strains.^1^ Though the incidence and severity of annual influenza epidemics are greatly reduced by vaccines covering the circulating strains, influenza remains a major public health issue. Seasonal influenza vaccines are effective only against strains closely related to the vaccine strains, and do not protect against genetically drifted or influenza viruses newly introduced in the human population, resulting in a steep drop of vaccine effectiveness when vaccine strains are mismatched with circulating strains.^2,3^ The 2009 swine flu pandemic and newly emerging influenza strains with pandemic potential, such as H5N1 and H7N9, underscore the need for more broadly protective influenza vaccines.

The identification of broadly neutralizing monoclonal antibodies (bnAb)^4,5^ has fueled efforts to develop broadly protective influenza vaccines, able to elicit these types of antibodies. The majority of these bnAb bind specifically to the membrane-proximal “stem” region of the hemagglutinin (HA) protein. In contrast to the variable HA head region, the stem region of the HA protein is highly conserved.^6^ However, antibodies targeting the stem are found only in low frequency in humans after vaccination or infection with a seasonal influenza strain.^7–9^ Various approaches to induce a potent immune response against the less immunogenic stem region of the HA protein were tested in preclinical animal models such as sequential infections,^10^ sequential immunizations with chimeric HA molecules,^11^ and shielding of the HA head epitopes by hyperglycosylation.^12–14^

A recently successful approach is removal of the immunodominant head region of the HA protein while maintaining the structure of the stem region on nanoparticles or by introducing stabilizing mutations.^15,16^ The group I mini-HA stem antigen described by Impagliazzo et al. has been shown to be immunogenic and to induce a cross-protective immune response in influenza-naive mice and non-human primates (NHP). However, prior exposure to influenza can profoundly affect the immune response to subsequent influenza infection and the protective efficacy of vaccination.^17^ Importantly, virtually all humans have detectable antibodies to at least one strain of influenza virus by the age of 6 years.^18^

To assess the impact of previous exposure to influenza on the induction of broadly influenza reactive antibodies by a mini-HA antigen, we used a cohort of NHP previously vaccinated and exposed to H1N1 influenza virus.^16^ The NHP were immunized with either a seasonal trivalent influenza vaccine (TIV) or group I mini-HA 3 months after H1N1 infection. We show that mini-HA immunization induces antibodies in these pre-exposed NHP binding to all group 1 influenza viruses tested, and that these antibodies bind to the stem region of the HA protein. We used an adoptive transfer mouse model^19^ to assess the protective efficacy of NHP antibodies against lethal group 1 influenza virus challenges. We show that vaccination of pre-exposed NHP with mini-HA induces an immune response which protects mice from lethal heterologous and heterosubtypic challenge. In accordance with these results, we found significant in vitro neutralization and ADCC titers of H5N1 influenza by immunization with mini-HA, but not the seasonal TIV.

## Results

**Fig. 1:**
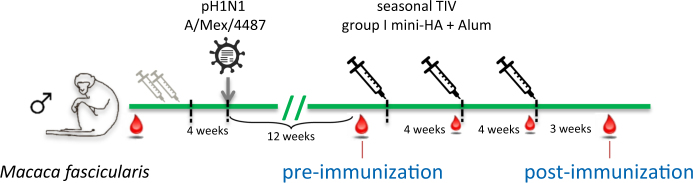
Previously exposed NHPs received three immunizations of either a human dose of seasonal TIV or mini-HA adjuvanted with Alum. Blood samples were collected at the beginning of the study, 1 day prior to each vaccination, and 3 weeks after the last vaccination

The cohort of NHP (*n* = 11) used for the current study was previously exposed to influenza infection, which in some animals was preceded by a TIV vaccination (*n* = 5), indicated by symbols in the figures. NHP were challenged with A/Mexico/InDRE4487/09 (A/Mex/4487) a pandemic H1N1 (pH1N1) A/California/07/09-like strain, homologous (>99% HA identity) to the TIV H1N1 component,^16^ see Fig. 1 for a schematic representation of the study design. In the current study, the NHP received three intramuscular (i.m.) immunizations with either TIV Inflexal V 2013/14 (human dose, *n* = 5) or group I mini-HA (150 µg, *n* = 6) adjuvanted with aluminum hydroxide (750 µg Alum), starting 3 months after challenge. NHP were allocated to either TIV or mini-HA immunization using a randomized block design, ensuring equal distribution of the animals with respect to their influenza pre-exposure history.

### Mini-HA induces binding antibodies to a broad panel of rHA

**Fig. 2:**
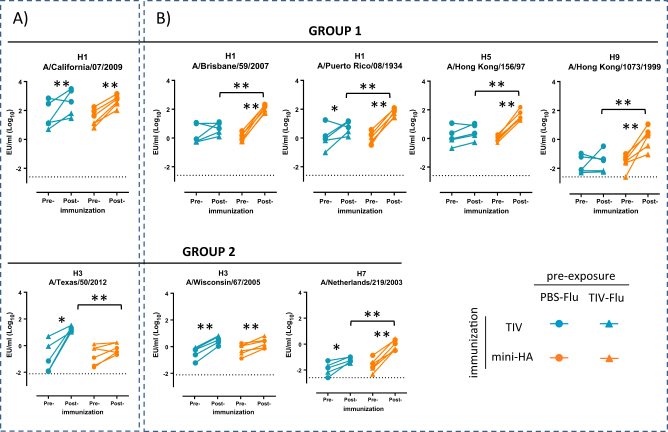
Group I mini-HA induces binding antibodies to a broad panel of rHAs in pre-exposed NHP. Binding antibody titers 1 day prior to immunization (pre-) and 3 weeks post (post-) the third immunization. **a** ELISA titers of the H1 and H3 influenza strains included in the TIV. **b** ELISA titers of the group 1 and 2 rHA not included in the TIV. Symbols indicate different pre-exposure histories. Solid lines indicate paired observations. Dashed lines indicate LOD. All baseline naive serum samples taken prior to challenge are on LOD (data not shown). Comparisons are made between pre-immunization and post-immunization, and between mini-HA and TIV immunization per time point; *p < 0.05, **p < 0.01

To determine whether immunization of pre-exposed NHP with TIV and mini-HA could induce broadly binding antibodies, we compared binding to a panel of group 1 and group 2 influenza A virus HAs (see Fig. 2). Blood samples were taken prior to immunization and 3 weeks after the third immunization, and serum was isolated for analysis. As expected, TIV immunization induced a significant increase in antibody titers against the rHAs of the two influenza A strains included in the vaccine (H1 A/California/07/2009, *p* < 0.01, and H3 A/Texas/50/2012, *p* < 0.05; see Fig. 2a). Immunization with mini-HA also significantly increased binding to this H1 HA (*p* < 0.01), but not H3 (*p* = 0.068). Immunization with the mini-HA in addition significantly induced binding to all other group 1 rHAs tested (*p* < 0.01). Comparing post-vaccination titers of mini-HA to TIV-immunized animals we observed that mini-HA-induced titers were significantly (9–36-fold) higher (*p* < 0.01) (see Fig. 2b). Antibody binding titers to two group 2 influenza rHAs H3 A/Wisconsin/67/2005 (both immunogens *p* < 0.01) and H7 A/Netherlands/219/2003 (*p* < 0.05 by TIV and *p* < 0.01 by mini-HA immunization) were significantly higher after both TIV and mini-HA immunization as well, although this increase appears to be less pronounced compared to group 1 HA antibody titers. The antibody titers binding to the rHA H7 were significantly higher after immunization with mini-HA compared to the TIV (*p* < 0.01).

Although we cannot statistically test for significance due to small group sizes, in most enzyme-linked immunosorbent assay (ELISA) it appears that TIV immunization during pre-exposure resulted in slightly lower titers compared to the group not immunized during pre-exposure (“PBS-Flu” group), in particular at the pre-immunization time point. This is most likely due to differences in influenza infection after challenge, which was reduced in the TIV pre-exposed group.^16^

### Mini-HA boosts HA stem-binding antibodies

**Fig. 3:**
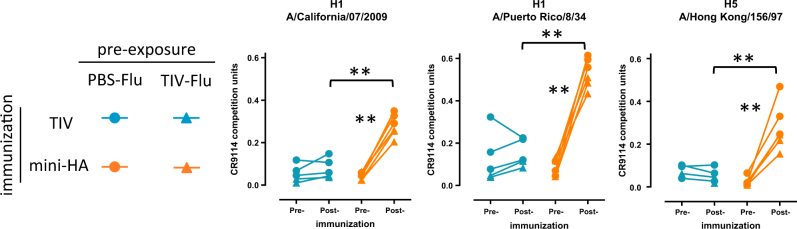
Mini-HA boosts CR9114 competing HA antibodies. Serum taken 1 day prior and 3 weeks post-immunization were tested for competition with bnAb CR9114, using H1 A/California/07/09, H1 A/PuertoRico/8/34, or H5 A/HongKong/Vietnam fusion as antigen. Symbols indicate different pre-exposure histories. Comparisons were made between TIV and mini-HA immunization; **p < 0.01

We then wanted to confirm that the different vaccination regimens induce antibodies targeting different regions of the HA protein, in particular the receptor binding site (RBS) at the head region in contrast to the stem region. Sera taken 1 day prior to the first immunization and 3 weeks after the third immunization were tested for antibodies binding near the RBS using the haemagglutination inhibition (HI) assay. As expected, immunization with TIV but not the mini-HA significantly increased HI titers against pH1N1 A/California/07/09, the H1N1 strain included in the seasonal vaccine (see Supplementary Fig. 1). These haemagglutination inhibiting antibodies are likely specific for the pH1N1 strain as no HI titers were detected against A/Puerto Rico/8/34, a heterologous H1N1 influenza strain. In contrast, we found a significant induction of antibodies competing with stalk-specific bnAb CR9114 for binding to HA from A/California/07/09 after immunization with mini-HA, but not TIV (Fig. 3). When repeated with HA’s derived from other group 1 strains similar results were obtained, even though both vaccines contain the stem region of H1 HA.

### Passive antibody transfer from mini-HA vaccinated, pre-exposed NHP protect mice against lethal H1N1 and H5N1 challenge

HA stem-specific antibodies prevent influenza by fundamentally different mechanisms not detected by HI.^20^ Therefore, we wanted to determine whether the stem-specific antibodies induced by mini-HA would be able to protect against lethal group 1 influenza challenges. For this, we used an adoptive transfer mouse model previously described for testing of human serum samples.^19^ Mice received an intraperitoneal injection with NHP serum and plasma. Samples from the pre-exposed NHP were taken prior to immunization or 3 weeks after the third immunization. Naive control samples were taken before any immunization and challenge.^16^ Subsequently the mice were challenged 1 day later with either H1N1 A/Puerto Rico/8/34, an influenza strain heterologous to the pH1N1 NHP challenge and TIV strain, or H5N1 A/Hong Kong/156/97, a strain genetically more distant and heterosubtypic to pH1N1.

**Fig. 4:**
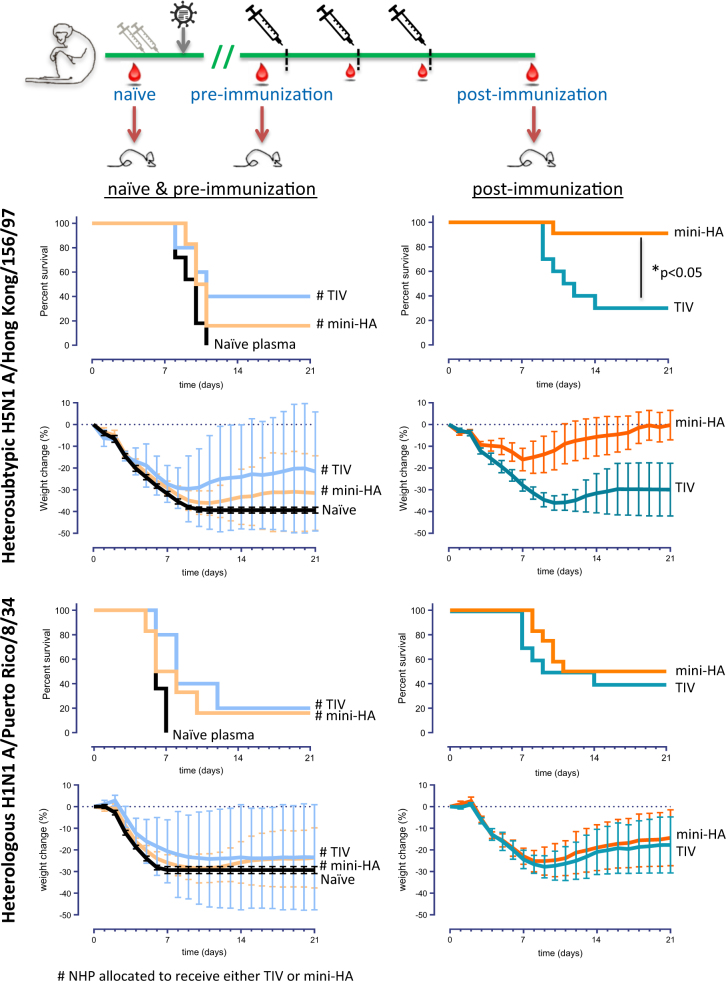
NHP serum and plasma protect mice from lethal influenza challenge. Mice received an intraperitoneal injection of 400 µl with serum and plasma from NHP and were subsequently inoculated 1 day later with 12.5× LD50 of either H5N1 A/Hong Kong/156/97 (top panels) or H1N1 A/Puerto Rico/8/34 (bottom panels) influenza virus. Naive plasma samples taken prior to treatment of the NHP were used as control (left panels). Serum and plasma samples were taken 3 months after virus inoculation and 1 day prior to immunization (left panels) or 3 weeks after the third vaccination (right panels). Kaplan–Meier curves show the survival percentage per vaccination regimen indicated alongside each line post-challenge (d0) until end of follow-up (day 21). Mean relative bodyweight change per vaccination regimen post-challenge (d0) until end of follow-up (day 21) are shown. Bodyweights are expressed relative to weight on day 0. Error bars indicate 95% CI (mean ± 1.96 × standard deviation)

Transfer of NHP serum and plasma samples of the pre-immunization time point showed partial protection compared to transfer of serum from naive non-immunized control time points in both murine challenge models, probably due to the pre-exposure history of the NHP (Fig. 4, left panels). In the H5N1 A/Hong Kong/156/97 challenge model, NHP immunization with mini-HA induced a protection of 91%, significantly higher compared to NHP immunization with TIV (*p* < 0.05, Fig. 4, right panels). These results show that immunization of pre-exposed NHP with the mini-HA elicits a humoral immune response which is cross-protective against lethal influenza challenge when transferred to mice.

In the H1N1 A/Puerto Rico/8/34 challenge model we found partial protection by both vaccination regimens, albeit it not significantly increased relative to plasma and serum transferred from the pre-immunization time point. The H1N1 A/Puerto Rico/8/34 influenza virus appears to be a relatively virulent challenge strain in mice, as can be deducted from the relatively short time of death of the control group (i.e., within 7 days, compared to 11 days after H5N1 challenge). In addition, NHP antibody titers are diluted 10-fold by the transfer to mice. To increase antibody titers in mice challenged with H1N1 A/Puerto Rico/8/34, and approximate titers prior to transfer, mice received NHP plasma and serum on 3 consecutive days. Although in total more mice were protected from challenge, again both mini-HA and TIV samples protected mice to comparable levels (75 and 60%, respectively) (see Supplementary Fig. 2).

### Mini-HA immunization induces heterosubtypic H5 neutralization and hFcγRIIIa signaling in pre-exposed NHP

**Fig. 5:**
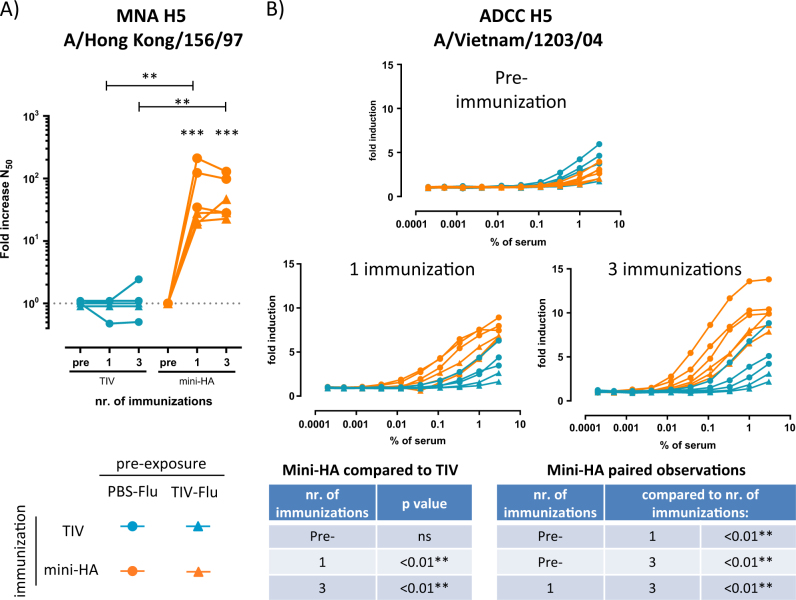
Mini-HA boosts H5N1 neutralization and hFcγRIIIa signaling. Serum taken 1 day prior, 4 weeks after the first immunization, and 3 weeks after the third immunization (previously denoted as “post”) were tested for **a** fold increase in H5N1 neutralization (fold increase in N50, solid lines indicate paired observations) and **b** hFcγRIIIa activation in a surrogate H5N1 ADCC assay. Symbols indicate different pre-exposure histories. Three MNA samples in the TIV group were on LOD and were nudged for visualization. Statistical comparisons are made between paired observations per immunization regimen and between mini-HA and TIV immunization (indicated with asterisks in the MNA figure or ADCC tables); ns not significant, **p < 0.01, ***p < 0.001

We subsequently investigated whether the difference in protective efficacy after the transfer of pooled serum and plasma transfer from NHP immunized with mini-HA compared to immunization with TIV observed in the H5N1 mouse challenge model is associated with antibody-mediated effector mechanisms. Therefore, we tested serum samples taken before and after immunization of the pre-exposed NHP for in vitro influenza virus neutralization and antibody-dependent cellular cytotoxicity (ADCC) activity, using an ADCC reporter assay measuring hFcγRIIIa signaling. Sera from pre-exposed NHP immunized three times with mini-HA showed a significant increase of H5N1 neutralization after immunization (*p* < 0.001) (see Fig. 5a). These titers are significantly higher than those induced by three immunizations with TIV (*p* < 0.01), in line with the observed protection of mice. In addition, we tested whether one immunization of pre-exposed NHP with mini-HA would induce detectable neutralization. As shown in Fig. 5a, significant H5N1 neutralization titers were detected after one immunization (*p* < 0.001), with similar levels compared to three immunizations. The ADCC reporter assay showed that mini-HA immunization significantly induced hFcγRIIIa signaling after only one immunization (*p* < 0.01), which increased after three immunizations (one compared to three immunizations; *p* < 0.01) (see Fig. 5b). Moreover, the ADCC titers observed were significantly higher than the titers induced by TIV immunization (*p* < 0.01).

## Discussion

Virtually all adults have been exposed to influenza multiple times and such an exposure can have a profound effect on the immune response to vaccination. Each encounter with influenza by infection or vaccination shapes the immune repertoire, and the potential induction of broadly influenza reactive antibodies will be modulated by the existing immunological memory. Therefore, it is key for the development of a universal HA stalk-based vaccine to determine whether cross-reactive antibodies can be induced in the presence of influenza-specific immunity. In addition to the previously described broad protection in naive animal models, we here show that a group 1 mini-HA induces broadly influenza reactive antibodies in influenza experienced NHP. Accordingly, serum from these mini-HA immunized NHP conferred partial protection against heterologous H1N1 challenge and superior protection compared to a seasonal TIV against heterosubtypic H5N1 challenge in mice. The protection against H5N1 induced by mini-HA appeared to be associated with functional antibodies that can either directly neutralize the virus or trigger effector mechanisms.

As humans are exposed to influenza their immune response to new influenza infections or vaccination is being shaped.^21^ Most influenza neutralizing antibodies are directed to the highly variable immunodominant HA head region.^22^ However, unlike this strain-specific and dominant HA head response induced by seasonal drifted strains, first exposure to an antigenically different influenza strain predominantly boosts a response of cross-reactive memory B cells which target the HA stem region.^8,23–25^ Unfortunately this broadly protective immune response is found only transiently and re-exposure to antigenically similar influenza strains will focus the immune response again to immunodominant HA head epitopes.^19,26^ In agreement with this, our results show that immunization of pre-exposed NHP with a seasonal TIV potently induced antibodies directed to the globular head domain of the HA molecule while no increase in antibodies which compete for a broadly neutralizing stem epitope was detected. In addition, even though H5N1 neutralizing antibodies could be detected in some NHP after exposure to H1N1 influenza, these titers were not enhanced after subsequent immunization with TIV.

Various approaches to avoid the immunodominant HA head response and induce a potent immune responses against the stem region of the HA protein have been tested.^11,13,27,28^ Recently, we have shown the design of a headless group I mini-HA by the introduction of stabilizing mutations to be immunogenic and induce a cross-protective immune response in influenza-naive animal models.^16^ Here we show that immunization of pre-exposed NHP with this group I mini-HA potently induced cross-reactive antibodies binding to a panel of group 1 rHAs. Moreover, as previously observed in naive NHP, the mini-HA in addition induced statistically significant higher antibody titers binding to group II H7 HA than TIV. We intend to explore this in future studies as the explanation for this is still unclear. Looking into the HA target region of the antibodies induced by the vaccines we found that in contrast to TIV, mini-HA induced antibodies in pre-exposed NHP specifically targeting the HA stem region and thereby competing for a broadly neutralizing stem epitope.

Antibody titers appeared to be slightly lower in NHP that received TIV in the pre-exposure history prior to the influenza challenge. The difference appeared to be larger at the pre-immunization time point and vaccination with both TIV and mini-HA seems to reduce the difference. Although it is difficult to draw conclusions due to the small number of animals in each group, it is most likely that TIV immunization preceding the pH1N1 virus infection reduces the infection and hence does not optimally prime the animals for subsequent immunization with TIV and mini-HA. It has been described in literature that vaccination prior to infection reduces the cross-protective response.^29,30^ While in these studies the heterosubtypic protection is attributed to T cells, which do not play a role in these serum transfer studies, it might be that in our study the quality of the antibodies is reduced due to suboptimal priming of the HA-specific immune response prior to vaccination.

Binding of antibodies to conserved stem epitopes, however, does not provide any functional information on the protective capacity in vivo. Therefore, we used an adoptive transfer mouse model previously established to test human serum samples for protective efficacy,^19^ to test whether immunization of pre-exposed NHP would induce an in vivo cross-protective immune response. We found partial heterologous and heterosubtypic protection by serum and plasma of pre-exposed NHP prior to immunization. These results confirm other studies which observed cross-reactive antibodies in various animal models and humans after prior infection or vaccination.^8,19,23,31–33^ Subsequent immunization of pre-exposed NHP with both the mini-HA and TIV appear to boost this protective immune response against H1N1 A/Puerto Rico/8/34. However, not all mice were protected by the subsequent immunizations of the pre-exposed NHP, as serum antibody titers after a single transfer did not reach the same level as titers induced after active immunization (data not shown). In a separate follow-up study the number of transfers was increased (3 × 400 µl) to approximate antibody titers found in the pre-exposed NHP,^19^ which resulted in increased protection for both TIV and mini-HA immunized groups with a similar pattern as observed after 1 × 400 µl. In addition, the observation that all mice which received naive NHP plasma succumbed to the infection within a week suggests this is a stringent influenza challenge model. Differences in protection between H1 mini-HA and TIV immunized groups against this strain could therefore be difficult to detect, which might be overcome by increasing the number of mice or NHP or reducing the virus challenge dose. In contrast, 90% of mice which received samples from pre-exposed NHP immunized with mini-HA were protected against lethal heterosubtypic H5N1 A/Hong Kong/156/97 challenge, a strain genetically more distant from the H1N1 used for pre-exposure of the NHP. Immunization with TIV on the other hand did not induce a protective humoral immune response against H5N1 influenza. The absence of significant H5N1 protection by TIV vaccination is in line with a previous study in which samples from healthy human adults immunized three times with TIV did not confer significant protection against H5N1 influenza in mice.^19^ Together, these results show the potential benefit of cross-protection induced by mini-HA vaccination over a seasonal TIV.

Currently the only established in vitro correlate of protection by influenza vaccines is blocking of viral attachment to host cells as measured by the HI assay. Although this correlate has been proven valuable to determine vaccine effectiveness of vaccines containing the intact HA protein, HA stem-specific antibodies neutralize influenza viruses by fundamentally different mechanisms not detected by HI.^20^ Potential mechanisms of neutralization include blocking of viral entry, interfering with the fusion of viral and host membranes, inhibiting HA0 cleavage and Fc receptor-dependent pathways.^34^ In addition, compared to antibodies binding to the HA head, stem-specific antibodies were shown to have a lower neutralizing capacity in vitro.^35^ Taken together, defining a correlate of protection for HA stem-specific antibodies has proven to be difficult. Here we show that the cross-protection induced by mini-HA but not TIV is associated with a significant in vitro induction in neutralization of the H5N1 challenge strain. We did not detect H1N1 A/Puerto Rico/8/34 virus neutralization titers (data not shown), which might correspond with observed lower protective efficacy. Interestingly, one immunization with the mini-HA induced high H5N1 neutralization titers which do not further increase after multiple immunizations, which has also been observed for CR9114 competing antibody titers (data not shown). This suggests that one immunization with the mini-HA may be sufficient for protection against H5N1 in a pre-exposed setting. Moreover, and as expected, these results confirm that the mini-HA can be used for multiple immunizations of pre-exposed NHP without the generation of immunity to the HA head region in line with studies performed in pre-exposed mice and ferrets.^32^ Besides in vitro neutralization, several studies have shown that certain cell-mediated effector functions play a role as well in protection mediated by stem-specific antibodies.^36–38^ In humans, effector functions such as ADCC and ADCP are potently induced by activation of the hFcγRIIIa (CD16) receptor.^39,40^ Our results show that the antibodies induced by Alum-adjuvanted mini-HA immunization significantly activated this receptor in NHP previously exposed to influenza. In agreement with a study in humans, significantly less signaling of the hFcγRIIIa receptor was induced by immunization with (unadjuvanted) TIV.^19^ The lack of hFcγRIIIa activation by antibodies induced by TIV makes it unlikely that these antibodies induce ADCC and ADCP.

The fact that the mini-HA is also immunogenic in pre-exposed NHP and a single immunization induces high H5N1 neutralization confirms the potential of a mini-HA-based universal influenza vaccine to boost a stem-specific cross-reactive immune response. However, the immune history to antigen exposure in humans is often more complex than in NHP, with successive influenza infections and vaccinations shaping the immune response. Because it will be impossible to recapitulate the complex human pre-existing immunity against influenza in an animal model, clinical trials will be essential to test whether the mini-HA is a viable universal influenza vaccine. In addition to immunological readouts, serum transfer may be a valuable tool to evaluate the cross-protective capacity of universal influenza vaccine candidates during clinical development.^33^

In conclusion, we show here the ability of mini-HA to elicit a broad and protective immune response in NHP previously exposed to influenza. Our results show that compared to a seasonal TIV, mini-HA immunization induced significantly higher titers of stem-specific antibodies binding to a panel of group 1 influenza viruses. When transferred to mice, antibodies induced by both immunizations improved protection against lethal heterologous H1N1 influenza challenge, although the difference in survival failed to reach significance. The mini-HA showed an additional benefit of protecting mice against lethal heterosubtypic H5N1 influenza challenge, whereas TIV vaccination did not. In addition we have shown that H5N1 survival is associated with H5N1 neutralization and H5 HA-specific ADCC titers. Taken together, these results show that a mini-HA-based vaccine should be explored further in pre-exposed populations and may eventually function as a seasonal vaccine with increased coverage against genetically drifted strains, and potentially against emerging strains.

## Methods

Unless stated otherwise, all experiments shown have been performed once in the laboratory. All research described in this paper was conducted in accordance with all relevant guidelines and procedures. The work has been approved and conducted in accordance with the European guidelines (EU directive on animal testing 86/609/EEC) and local Dutch legislation on animal experiments. The execution of this research was approved by the Biomedical Primate Research Center Animal Welfare Body (AWB or Instantie voor Dierenwelzijn, IVD) in accordance with Dutch law, and Janssen Vaccines & Prevention BV.

### Pre-exposure of NHP, immunization, and transfer experiments to mice

#### Pre-exposure

A cohort of NHP were previously exposed to influenza (see Impagliazzo et al.).^16^ Briefly, male cynomolgus macaques (*Macaca fascicularis*) were pre-screened and found negative for serum antibodies against alpha herpes virus, Simian T-cell Leukemia virus, Simian Retro Virus, influenza A Nucleoprotein, and HI against the challenge virus. Animals were randomly allocated to groups of six animals each. One group received two i.m. immunizations with the human dose (0.5 mL) of TIV Inflexal^®^ V season 2013/14 (Crucell, Bern, Switzerland). Another group received three times 0.5 mL phosphate buffered saline (PBS) i.m. The immunizations were performed with a 4-weeks interval. Four weeks after the final immunization animals were challenged intrabroncheally with 4 × 10^6^ 50% Tissue Culture Infective Dose (TCID_50_) H1N1 A/Mexico/InDRE4487/2009.

#### Immunization

Each pre-exposure group was split and allocated to either receiving TIV or mini-HA (UFV4900, PDB ID 5CJQ) immunizations using a randomized block design taking pre-exposure history into account. Twelve weeks after challenge five NHP received three i.m. immunizations with a human dose (0.5 mL) of TIV Inflexal^®^ V season 2013/14 (containing among others A/California/7/2009 (H1N1pdm09), homologous to H1N1 A/Mexico/InDRE4487/2009). Six NHP received three i.m. immunizations with 150 µg mini-HA UFV4900 protein adjuvanted with 750 μg alum (2% Alhydrogel, Brenntag) in a total volume of 500 µL. Because comparison of groups with group sizes smaller than five animals lacked the statistical power for analysis, the NHP were grouped irrespective of the different pre-exposure histories.

#### Serum transfer

Serum and plasma from NHPs were isolated and transferred (400 μL, intraperitoneally) to naive recipient mice prior to challenge, per NHP one mouse received plasma and one mouse received serum, thus there were two observations per NHP. All samples were blinded during the experiment and were unblinded during analysis, after the experiment finished and results had been quality controlled. The NHP was the experimental unit for statistical analysis, so first the serum and plasma data was summarized per NHP and only then standard statistical methods could be applied. Across all groups (*n* = 11 NHP), we could not detect any statistical significant difference between survival after transfer of serum compared to plasma transfer (*p* = 1.000 for both the H1N1 PR8 and H5N1 HK97 challenge strain). This confirms that serum and plasma datasets can be pooled, to increase the accuracy of the observation per NHP, and thereby gaining enough statistical power for analysis. As negative controls, mice received plasma from naive NHP taken before treatment. The absence and presence of full-length (FL) HA-specific antibodies in recipient mice after transfer of serum was confirmed by ELISA. As observed previously, antibody titers dropped on average 10× when transferred to mice. As an established positive control for the challenge experiments, bnAb CR6261 was administered at 15 mg/kg intravenously 1 day before the challenge. Mice were challenged intranasally with 25 50% Lethality Dose (LD50) of influenza virus under ketamine/xylazine anesthesia. After challenge, mice were monitored for survival, bodyweight loss, and clinical score for up to 21 days. In addition, 400 µl of NHP serum and plasma were transferred on 3 consecutive days and subsequently challenged with 25 LD50 of H1N1 A/Puerto Rico/8/34 and monitored for 21 days.

### Enzyme-linked immunosorbent assay

Antibody responses against selected FL HA were obtained by ELISA according to previous described methods.^16^ Briefly, 96-well plates were coated with 100 µL of 0.5 μg/mL recombinant FL HA (Protein Sciences, Meriden, CT). Plates were washed with PBS-Tween (PBS-T) and subsequently blocked with block buffer. After washing, serum or plasma were randomly added to the plate in duplicate, serially diluted in block buffer and incubated. Following a wash with PBS-T, a 1:2000 dilution of Goat-anti-Mouse IgG-HRP was added to the plate for mouse samples, or a 1:5000 dilution of Goat-anti-Monkey IgG-HRP for macaque samples.

Plates were washed and developed using o-phenylenediamine dihydrochloride (OPD) substrate. The colorimetric reaction was stopped after 10 min by adding 1 M H_2_SO_4_ and the optical density (OD). The OD of each sample dilution was then quantified against the standard curve included on each plate, consisting of a murine IgG2a version of CR9114 for mouse samples or CR9114 for macaque samples, and the final concentration per sample calculated by a weighted average, using the squared slope of the standard curve at the location of each quantification as weight. Negative samples were set at the limit of detection (LOD), defined as the lowest sample dilution multiplied by the lowest standard concentration with an OD response above the lower asymptote of the standard curve and background. ELISA titers were expressed as log10 ELISA Units (EU) per ml.

### CR9114 competition ELISAs

Antibodies competing with CR9114 over a broadly neutralizing epitope on the HA stem were determined as previously described.^16^ Briefly, plates were coated with purified polyclonal rabbit anti-His-Tag IgG followed by washing. After blocking and washing, plates were incubated with His-tagged FL HA. Plates were washed and serum samples were added randomly over groups to the plates in duplicate, serially diluted in block buffer. Subsequently a titrated amount of biotinylated human IgG1 CR9114 was added (0.001 µg for H1 A/Brisbane/59/07 and A/California/07/09 and 0.0015 µg for H5 A/Hong Kong/156/97) and incubated. After washing, streptavidin-horse radish peroxidase (HRP) was added and incubated, followed by washing and OPD development. The OD was measured and standard curves were created using a four-parameter logistic curve. The CR9114 competition of each sample was quantified as the slope of the linear regression of OD value on the log10 dilution for the duplicate series.

### HI assay

HI assay was performed as described before.^16^ Shortly summarized, non-specific agglutination inhibitors were removed from serum samples by incubation with *Vibrio cholerae* neuraminidase which was subsequently inactivated by incubation with sodium citrate. Turkey red blood cells diluted in PBS were added, incubated, and subsequently spun down. Two-fold serial dilutions of the supernatant in PBS were prepared in duplicate, mixed by agitation with four HA units wild-type viruses H1N1 A/California/07/2009 or H1N1 A/Puerto Rico/8/34, and incubated followed by addition of turkey red blood cells. Plates were again incubated and the haemagglutination status of each well was visually determined. The assay titer of a given serum sample was defined as the reciprocal of the highest dilution where no HI was observed.

### Microneutralization assay

The MNA assay was performed as previously described.^16^ Briefly, Madin–Darby Canine Kidney (MDCK, negative for mycoplasma) cells were seeded in 96-well plates, at 15,000 cells per well in assay medium and allowed to attach for a minimum of 3 h. Duplicate serial dilutions of heat inactivated NHP serum samples were prepared in assay medium without trypsin/EDTA and mixed with H5N1 reassortant A/Hong Kong/156/97 (rgPR8-H5N1 6:2 reassortant, containing HA and NA from H5N1 A/Hong Kong/156/97) virus in assay medium containing trypsin/EDTA. This mix was incubated at 37 °C for 1.5 h. Virus and serum were subsequently added to the MDCK cells at a final concentration of 100 TCID50 virus per well and incubated for 20 h. Cells were fixed with acetone and air dried. Plates were washed with PBS-T and labeled with biotin-conjugated mouse anti-influenza A nucleoprotein for 1 h. After washing with PBS-T, plates were incubated with Streptavidin-HRP for 1 h, washed with PBS-T, and 2,2′-azino-bis (3-ethylbenzothiazoline-6-sulfonic acid) (ABTS) substrate added. Absorbance was read using a BioTek^®^ reader after 45 min. The N_50_ values were calculated after 4-parameter logistic curve fit. Fold increase of the N_50_ were calculated compared to baseline serum samples (pre-immunization).

### ADCC reporter assay

Signaling by the hFcγRIIIa activation as measured in the ADCC reporter assay was performed as previously described.^16^ Briefly, human lung carcinoma-derived A549 epithelial cells (ATCC, <10 passages, tested for mycoplasma) were maintained in Dulbecco’s Modified Eagle Medium supplemented with fetal calf serum. Two days before the experiment, cells were transfected with plasmid DNA encoding H5 FL HA A/Vietnam/1203/04. One day before the assay, transfected cells were harvested and seeded in 96-well plates. After 24 h, samples were diluted in assay buffer and heat inactivated, followed by serial dilution in assay buffer. The cells were replenished with fresh assay buffer and ADCC Bioassay effector cells (a stable Jurkat cell line expressing human hFcγRIIIa (V158 variant)), human CD3γ, and an NFAT-response element driving expression of a luciferase reporter gene were added and incubated. Bio-Glo Luciferase Assay System substrate was added and Luminescence was read. Data are expressed as mean fold change of RLU per serum concentration relative to the RLU in absence of serum.

### Statistical analysis

Statistical differences between immunization with mini-HA and TIV were evaluated for HA-specific binding antibodies, CR9114-competing antibodies, ADCC reporter assay, and in the MNA assay. Data were log-transformed except for the competition ELISA and ADCC assay. Comparisons between the immunizations were made using the exact Wilcoxon rank-sum test. Additionally, the effect of three immunizations compared to one immunization, or the effect of immunization compared to pre-immunization samples, was determined per immunization regimen using the one-sample *t*-test.

In the serum transfer and mouse challenge studies, a study was considered valid only when there was a statistically significant difference in survival proportion (Fisher’s exact test, two-sided) between negative (mock) and positive (mAb CR6261, administered 1 day prior to challenge) challenge model control groups. The comparison between mini-HA and TIV for survival proportion is made with the Wilcoxon rank-sum test on a summary score per NHP as experimental unit. Per NHP one mouse received plasma and one mouse received serum. The survival of these two mice after challenge is summarized as a score of either 0/2, 1/2, or 2/2 and used in the comparison.

Statistical analyses were performed using SAS version 9.4 (SAS Institute Inc., Cary, NC, USA) and SPSS version 20 (SPSS Inc., IL, USA). Statistical tests were conducted two-sided at an overall significance level of *α* = 0.05.

### Data availability

The data that support the findings of this study are available from the corresponding author upon reasonable request.

## Electronic supplementary material

Supplementary figures 1 and 2
