# Supplementary material for: Mini-hemagglutinin vaccination induces cross-reactive antibodies in pre-exposed NHP that protect mice against lethal influenza challenge
Source: NPJ Vaccines. 2018 Jul 3;3:25. doi: 10.1038/s41541-018-0063-7 (PMC6030213; doi:10.1038/s41541-018-0063-7)
Supplement: Supplementary file 1 — Supplementary figures 1 and 2 [file 41541_2018_63_MOESM1_ESM.pdf]

Supplementary figure 1)

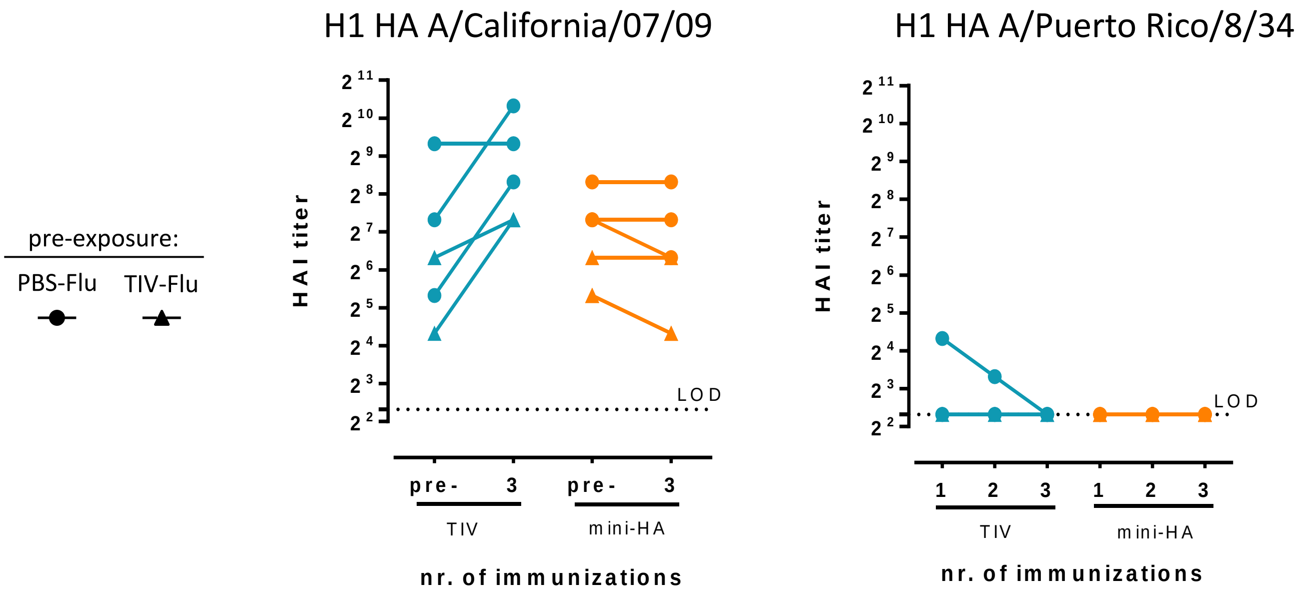

TIV induces homologous, but not heterologous HI titers .

Serum taken one day prior; “0”, or several weeks after immunization; “1”, “2” and “3”, were tested for H1N1 haemagglutination inhibition of A/California/07/09, the strain included in the seasonal TIV, and A/Puerto Rico/8/34, an heterologous strain. Symbols indicate different pre-exposure histories.

Supplementary figure 2)

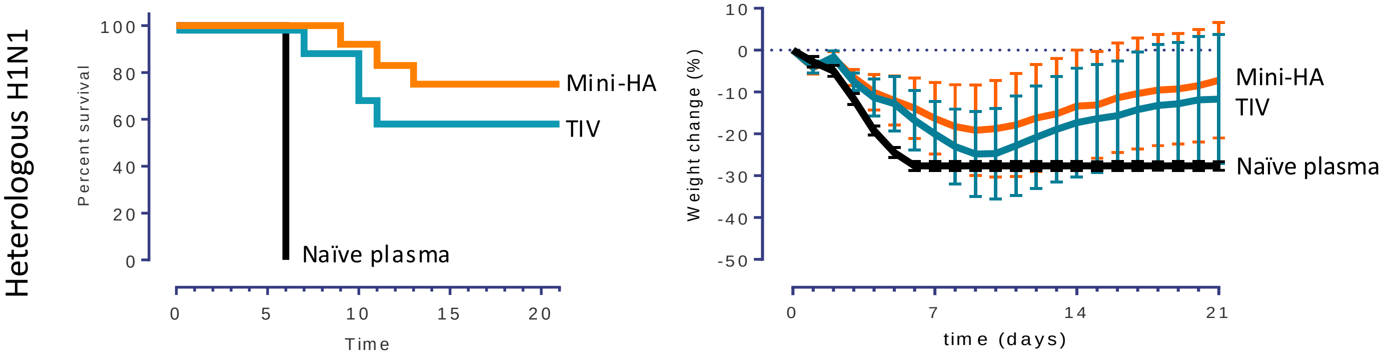

Multiple transfers of NHP serum and plasma protect mice from lethal H1N1 A/Puerto Rico/8/34 influenza challenge.

Serum and plasma samples from NHP were taken three weeks after the third vaccination ("post-immunization"). Naïve plasma samples taken prior to treatment of the NHP were used as control. Mice received an intraperitoneal injection of 400µl on three consecutive days and subsequently inoculated one day later with 12.5xLD_{50} H1N1 A/Puerto Rico/8/34 influenza virus. Kaplan-Meier curves show the survival percentage per vaccination regimen indicated alongside each line. Mean relative bodyweight change per vaccination regimen post-challenge (d0) until end of follow-up (day 21) are shown. Bodyweights are expressed relative to weight on day 0. Error bars indicate 95% CI (mean ± 1.96 x standard deviation).
